# Association between tea types and number of teeth: a cross-sectional study of the Chinese Longitudinal Healthy Longevity Survey

**DOI:** 10.1186/s12889-024-17874-7

**Published:** 2024-02-07

**Authors:** Zheqi Huang, Kahori Kawamura, Hideki Yoshimatsu, Tatsuro Miyake

**Affiliations:** 1 Graduate school of Dentistry, Osaka Dental University, 1-8 Kuzuha Hanazono-cho, Hirakata-shi, Osaka, 573-1121 Japan; 2 Department of Preventive and Community Dentistry, Osaka Dental University, 1-8 Kuzuha Hanazono-cho, Hirakata-shi, Osaka, 573-1121 Japan

**Keywords:** Tea type, Number of teeth, Chinese Longitudinal Healthy Longevity Survey, Older adults, Brushing frequency

## Abstract

**Background:**

Previous studies have suggested that tea consumption may have a positive impact on oral health. However, the effects of different tea types on oral health remain unclear. Therefore, this study aimed to determine the association between residual teeth and consumption habits of different types of tea (green tea, black tea, oolong tea, and scented tea) in older adults.

**Methods:**

We conducted a secondary analysis using data from the Chinese Longitudinal Healthy Longevity Survey in 2018. In a sample of 6,387 older adults, we performed logistic regression analysis to examine the relationship between persistent tea consumption and oral health according to sex and brushing frequency. The indices for particularly healthy oral health and relative health were set at more than 20 teeth and more than 10 teeth, respectively.

**Results:**

The study included 2,725 males and 3,662 females, both aged 65 and older. Among individuals with more than 20 teeth, drinking green tea significantly improved oral health in men (adjusted odds ratio [ORs]: 1.377; 95% confidence interval [CI]: 1.082–1.752) and drinking black tea significantly improved the oral health of women (ORs: 2.349, 95%CI: 1.028–5.366). In the daily brushing group, green tea had a significant beneficial effect on increasing the number of teeth in men and black tea had a significant beneficial effect in women. Among individuals with more than 10 teeth, drinking green tea significantly improved oral health in men (ORs: 1.539; 95% CI: 1.209–1.959) and drinking green tea and scented tea significantly improved the oral health of women (ORs: 1.447, 95%CI: 1.052–1.991; ORs: 1.948, 95%CI: 1.137–3.340). In the daily brushing group, consumption of green tea and black tea had significant beneficial effects on increasing the number of teeth in men, whereas that of green tea, black tea, and scented tea had significant beneficial effects in women.

**Conclusion:**

Long-term green tea consumption in males and black tea consumption in females were significantly associated with maintaining functional dentition (≥20 teeth). Similarly, long-term green tea consumption in males and green tea and scented tea consumption in females were associated with avoiding severe tooth loss (≥10 teeth). Furthermore, in the daily tooth brushing group, long-term consumption of black tea was associated with avoiding severe tooth loss in both sexes. However, tea consumption alone had no effect on oral health without good brushing habits.

## Background

According to the World Health Organization, tooth loss is the most common oral disease, affecting an estimated 1 billion people worldwide [1]. Tooth loss and its underlying mechanisms are widely associated with systemic diseases and nutritional status [2, 3], which have considerable effects on the overall health of older adults, resulting in enormous social and economic burdens [4]. The World Health Organization has proposed a goal of maintaining ≥20 teeth in adults aged 80 years, as a measure of good dental health [5]. Moreover, having <10 teeth is indicative of severe tooth loss, which seriously affects oral function [6].

Tea is among the most popular beverages worldwide and in China [7]. The three main types of tea are black tea (fully fermented), oolong tea (semi-fermented), and green tea (non-fermented). Another popular tea type is scented tea [8]. The consumption of green tea is correlated with oral health [9]. Specifically, catechins in green tea have been reported to have a bactericidal effect on *Streptococcus mutans*, *Candida albicans*, and *Porphyromonas gingivalis*, thereby improving oral microflora and periodontal conditions [10–13]. Similarly, theaflavins in black tea have antifungal effects against *C*. *albicans* [14]. Additionally, the components in oolong tea have been shown to exert antifungal effects against *C*. *albicans* [15]. Regarding scented tea, its extract has reportedly shown strong antibacterial effects against bacteria (*S*. *mutans* and* P*. *gingivalis)* in the oral cavity [16, 17].

Brushing teeth is important for oral health [18], and drinking tea can cause tooth discoloration, which may be eliminated by brushing or rinsing the mouth [19]. However, there are few studies on the oral health of people who are less aware of oral health and prefer drinking tea. There are significant sex-related differences in the association between tea consumption and cardiovascular disease [20, 21]. Accordingly, this study aimed to investigate the impact of different tea types on oral health according to sex and differences in tooth brushing frequency [9, 22]. We hypothesized that long-term tea consumption of various types would affect dental count in older adults differentiated by sex.

## Methods

### Study population

**Fig. 1:**
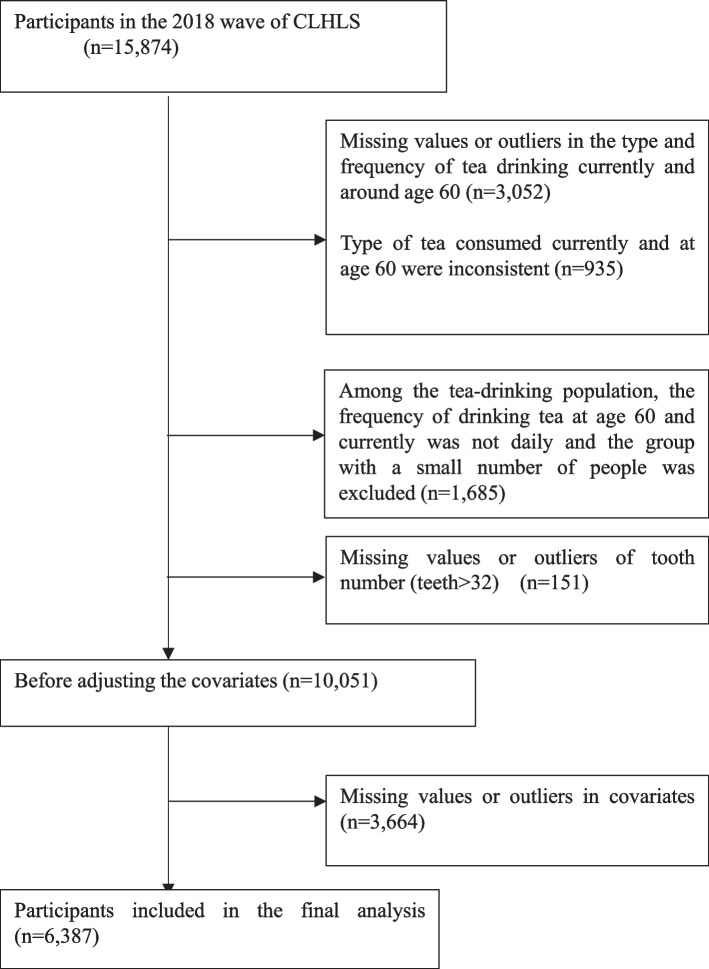
Derivation of the study population from the Chinese Longitudinal Healthy Longevity Survey (CLHLS) cohort

The data used for this study were obtained from the seventh wave of the Chinese Longitudinal Healthy Longevity Survey (CLHLS) dataset in 2018. The CLHLS conducted face-to-face interviews with centenarians (people aged 100 or above) randomly chosen across 23 Chinese provinces. For each centenarian, two local residents (same village, street, town, or city district) were also interviewed [23]. Predefined age and sex were randomly determined based on the assigned code numbers for matched comparisons of both sexes in each age group [7]. Interviews and basic physical assessments were conducted at participants' homes. Specifically, the survey covered diverse aspects of their lives, including demographics, family structure, diet, chronic diseases, healthcare, smoking and alcohol consumption, early nutrition, and health-related conditions. Refusal rates among the Chinese elderly were minimal due to their enjoyment of interaction and pride in their longevity [23]. For disabled individuals, proxy respondents (typically spouses or other close family members) were interviewed [23]. The survey included 15,874 older adults in 23 out of 31 Chinese provinces; among them, 67.4% were aged ≥80 years. The CLHLS study was approved by the Institutional Review Board of Duke University (Pro00062871) and the Biomedical Ethics Committee of Peking University (IRB00001052–13,074). The study protocol was approved by the Ethics Committee of Osaka Dental University (approval number: 111229; August 23, 2022), which waived the requirement for additional informed consent. Figure 1 presents the flowchart for patient selection. After excluding ineligible participants, 6,387 participants were included in the final analysis. Generally, we excluded outliers and missing values for the tooth number variable, the tea variable, and other covariates.

### Assessment of tooth loss

To collect information regarding tooth count, the survey asked respondents, “how many natural teeth does the respondent have (excluding false teeth)?” and “Do you have false teeth?” False teeth included both fixed and removable dentures in this study [24]. Based on previous reports, we categorized the number of teeth into two groups: more than 20 or less and more than 10 or less [5, 6].

### Exposure assessments

The items regarding the tea type were as follows: “What type of tea do you mainly drink at present?” and “What type of tea did you mainly drink around age 60?”. The responses regarding the frequency of tea drinking included the following: almost every day, not every day but at least once per week, not every week but at least once per month, not every month but occasionally, rarely or never. We selected people aged 60 years who were drinking the same type of tea almost every day. This was because the study focused on people aged >65 years who had consumed the same tea type for at least 5 years. The tea types included green tea, black tea, oolong tea, white tea, yellow tea, dark tea, compressed tea, scented tea, and others. Based on the sample size of the tea drinking population, we selected green tea, oolong tea, black tea, and scented tea.

### Covariates measurement

Age was divided into five groups: 65–69, 70–74, 75–79, 80–84, and ≥85 years. The participant characteristics included sex (male/female), ethnicity (Han/minority), residence (city/rural), and geographical region (northern China/eastern China/central China/south-western China). Educational level was divided into three categories according to years of education: 0, 1–6, and >6 years. Health level was classified as good, average, and poor. Household income was divided into five 20-percentile groups. Smoking habits were divided into three groups according to the number of years smoking as follows: never smoked, 0–14 years, and ≥15 years. Drinking habits were classified as never, current drinker, and former drinker. Marital status was assessed by providing a yes/no response to the following options: divorced, widowed, married but not living with a spouse, and never married. Brushing frequency was determined based on the daily frequency: less than once and once or more. Further, the presence of hypertension, diabetes, and cardiovascular disease was assessed based on a yes/no response. Body mass index (BMI) was classified based on the World Health Organization classification as follows: <18.5 kg/m^2^, 18.5–24.9 kg/m^2^, and ≥25 kg/m^2^ [25]. The frequency of sugar consumption was assessed based on the following responses: almost every day, at least once per week or month, rarely or occasionally. Activity of daily living (ADL) grades were also classified as disabled and non-disabled based on a previous study [26].

### Statistical analysis

Baseline characteristics are described according to the type of tea used. Continuous data are presented as mean ± standard deviation (SD) and categorical data as frequency with percentage. To determine the association between the type of tea and number of teeth, we set the target variable as two models with more than 20 teeth and 10 teeth or less. To calculate adjusted odds ratios (ORs) and 95% confidence intervals (CI), we performed multivariate analysis according to sex and brushing frequency with the following covariates: age, ethnicity, residence, geographical region, educational level, health, household income, smoking habit, drinking habit, marital status, hypertension, diabetes, BMI, cardiovascular disease, sugar frequency, and activities of daily living (ADL) capacity. Statistical significance was set at *p*<0.05, and all statistical analyses were performed using Stata version 17.0 (Stata Corp, College Station, TX, USA).

## Results

Table 1 summarizes the baseline characteristics by sex of the remaining 6,387 participants after excluding missing values and outliers among the 15,874 participants. A total of 2,725 men and 3,662 women accounted for 42.7% and 57.3% of the participants, respectively. Moreover, for man, 73.9% did not drink tea; among them, the mean number of remaining teeth was 10.7 ± 10.7. Additionally, 17.8%, 2.4%, 1.1%, and 4.8% of the total men consumed green, black, oolong, and scented tea, respectively, while the corresponding average number of remaining teeth was 14.7 ± 10.6, 16.0 ± 11.5, 12.7 ± 11.1, and 12.9 ± 11.1, respectively. For woman, 90.2% did not drink tea; among them, the mean number of remaining teeth was 8.9 ± 10.3. Additionally, 6.2%, 0.8%, 0.5%, and 2.3% of the total women consumed green, black, oolong, and scented tea, respectively, while the corresponding average number of remaining teeth was 13.0 ± 10.3, 16.1 ± 11.1, 10.6 ± 12.7, and 13.3 ± 10.6, respectively. In the male group, we found that in comparison to those who did not drink tea, those who had a higher ratio of tea consumption were younger older adults (65–69 years old), married, and resided in the city and had an education level >6 years, good health, a household income in the 5th quintile, a smoking habit of ≥15 years, a current drinking habit, a brushing frequency of once or more per day, a sugar frequency of almost every day, and no disability. In the female group, young and old people (65–69 years old), those with an education level >6 years, good health, a household income in the 5th quintile, who were married, and had a brushing frequency of once or more per day exhibited a higher ratio of tea consumption.

**Table 1:** Characteristics of the study participants by sex (%)

Variable		Male						Female					
	Subgroups	No tea	Green tea	Black tea	Oolong tea	Scented tea	Total sample	No tea	Green tea	Black tea	Oolong tea	Scented tea	Total sample
	Total sample, n (Average remaining teeth)	2,013	484	65	31	132	2,725	3,304	228	30	17	83	3,662
	%	73.9	17.8	2.4	1.1	4.8	100	90.2	6.2	0.8	0.5	2.3	100
Teeth	Number	10.7±10.7	14.7±10.6	16.0±11.5	12.7±11.1	12.9±11.1	11.7±10.8	8.9±10.3	13.0±10.3	16.1±11.1	10.6±12.7	13.3±10.6	9.3±10.4
Age	Years	83.0±10.7	79.9±10.3	76.7±9.4	78.4±10.2	78.7±9.2	82.0±10.7	85.4±12.1	82.1±11.6	79.1±11.1	82.4±14.5	81.1±11.9	85.1±12.1
Age group	Years												
	65–69	325 (16.1)	100 (20.7)	20 (30.8)	8 (25.8)	30 (22.7)	483 (17.7)	457 (13.8)	49 (21.5)	11 (36.7)	5 (29.4)	21 (25.3)	543 (14.8)
	70–74	263 (13.1)	100 (20.7)	16 (24.6)	6 (19.4)	25 (18.9)	410 (15.0)	397 (12.0)	26 (11.4)	1 (3.3)	2 (11.8)	14 (16.9)	440 (12.0)
	75–79	275 (13.7)	83 (17.1)	12 (18.5)	7 (22.6)	29 (22.0)	406 (14.9)	413 (12.5)	37 (16.2)	6 (20.0)	2 (11.8)	11 (13.3)	469 (12.8)
	80–84	318 (15.8)	63 (13.0)	5 (7.7)	2 (6.5)	13 (9.8)	401 (14.7)	412 (12.5)	36 (15.8)	5 (16.7)	3 (17.6)	7 (8.4)	463 (12.6)
	≥ 85	832 (41.3)	138 (28.5)	12 (18.5)	8 (25.8)	35 (26.5)	1,025 (37.6)	1,625 (49.2)	80 (35.1)	7 (23.3)	5 (29.4)	30 (36.1)	1,747 (47.7)
Ethnicity	Han	1,811 (90.0)	465 (96.1)	62 (95.4)	31 (100.0)	129 (97.7)	2,498 (91.7)	3,002 (90.9)	221 (96.9)	29 (96.7)	17 (100.0)	81 (97.6)	3,350 (91.5)
	Minority	202 (10.0)	19 (3.9)	3 (4.6)	0 (0.00)	3 (2.3)	227 (8.3)	302 (9.1)	7 (3.1)	1 (3.3)	0 (0.00)	2 (2.4)	312 (8.5)
Residence	City	1,121 (55.7)	325 (67.1)	41 (63.1)	19 (61.3)	87 (65.9)	1,593 (58.5)	1,749 (52.9)	144 (63.2)	15 (50.0)	12 (70.6)	60 (72.3)	1,980 (54.1)
	Rural	892 (44.3)	159 (32.9)	24 (36.9)	12 (38.7)	45 (34.1)	1,132 (41.5)	1,555 (47.1)	84 (36.8)	15 (50.0)	5 (29.4)	23 (27.7)	1,682 (45.9)
Geographical region	Northern China	415 (20.6)	103 (21.3)	22 (33.8)	5 (16.1)	71 (53.8)	616 (22.6)	745 (22.5)	36 (15.8)	16 (53.3)	2 (11.8)	52 (62.7)	851 (23.2)
	Eastern China	533 (26.5)	175 (36.2)	19 (29.2)	11 (35.5)	7 (5.3)	745 (27.3)	860 (26.0)	87 (38.2)	4 (13.3)	10 (58.8)	5 (6.0)	966 (26.4)
	Central China	481 (23.9)	110 (22.7)	10 (15.4)	4 (12.9)	5 (3.8)	610 (22.4)	803 (24.3)	81 (35.5)	3 (10.0)	1 (5.9)	7 (8.4)	895 (24.4)
	South-western China	584 (29.0)	96 (19.8)	14 (21.5)	11 (35.5)	49 (37.1)	754 (27.7)	896 (27.1)	24 (10.5)	7 (23.3)	4 (23.5)	19 (22.9)	950 (25.9)
Education level, years	0	538 (26.7)	56 (11.6)	9 (13.8)	2 (6.5)	21 (15.9)	626 (23.0)	2,153 (65.2)	90 (39.5)	9 (30.0)	12 (70.6)	31 (37.3)	2,295 (62.7)
	1–6	906 (45.0)	196 (40.5)	21 (32.3)	14 (45.2)	50 (37.9)	1,187 (43.6)	766 (23.2)	70 (30.7)	13 (43.3)	2 (11.8)	32 (38.6)	883 (24.1)
	>6	569 (28.3)	232 (47.9)	35 (53.8)	15 (48.4)	61 (46.2)	912 (33.5)	385 (11.7)	68 (29.8)	8 (26.7)	3 (17.6)	20 (24.1)	484 (13.2)
Health	Good	998 (49.6)	263 (54.3)	40 (61.5)	21 (67.7)	68 (51.5)	1,390 (51.0)	1,512 (45.8)	114 (50.0)	19 (63.3)	9 (52.9)	46 (55.4)	1,700 (46.4)
	Average	766 (38.1)	158 (32.6)	14 (21.5)	7 (22.6)	55 (41.7)	1,000 (36.7)	1,303 (39.4)	79 (34.6)	7 (23.3)	4 (23.5)	31 (37.3)	1,424 (38.9)
	Poor	249 (12.4)	63 (13.0)	11 (16.9)	3 (9.7)	9 (6.8)	335 (12.3)	489 (14.8)	35 (15.4)	4 (13.3)	4 (23.5)	6 (7.2)	538 (14.7)
Household income	1 (poor)	446 (22.2)	61 (12.6)	17 (26.2)	8 (25.8)	15 (11.4)	547 (20.1)	828 (25.1)	27 (11.8)	6 (20.0)	5 (29.4)	6 (7.2)	872 (23.8)
	2	453 (22.5)	75 (15.5)	6 (9.2)	7 (22.6)	22 (16.7)	563 (20.7)	756 (22.9)	36 (15.8)	8 (26.7)	1 (5.9)	17 (20.5)	818 (22.3)
	3	483 (24.0)	97 (20.0)	14 (21.5)	5 (16.1)	30 (22.7)	629 (23.1)	796 (24.1)	44 (19.3)	7 (23.3)	2 (11.8)	21 (25.3)	870 (23.8)
	4	263 (13.1)	98 (20.2)	11 (16.9)	3 (9.7)	30 (22.7)	405 (14.9)	382 (11.6)	42 (18.4)	4 (13.3)	1 (5.9)	23 (27.7)	452 (12.3)
	5 (wealthy)	368 (18.3)	153 (31.6)	17 (26.2)	8 (25.8)	35 (26.5)	581 (21.3)	542 (16.4)	79 (34.6)	5 (16.7)	8 (47.1)	16 (19.3)	650 (17.7)
Smoking habit	Never	1,020 (50.7)	145 (30.0)	16 (24.6)	8 (25.8)	46 (34.8)	1,235 (45.3)	3,090 (93.5)	212 (93.0)	28 (93.3)	14 (82.4)	71 (85.5)	3,415 (93.3)
	0–14 years	550 (27.3)	165 (34.1)	22 (33.8)	13 (41.9)	49 (37.1)	799 (29.3)	171 (5.2)	13 (5.7)	0 (0.0)	1 (5.9)	10 (12.0)	195 (5.3)
	≥15 years	443 (22.0)	174 (36.0)	27 (41.5)	10 (32.3)	37 (28.0)	691 (25.4)	43 (1.3)	3 (1.3)	2 (6.7)	2 (11.8)	2 (2.4)	52 (1.4)
Drinking habit	Never	1,177 (58.5)	232 (47.9)	29 (44.6)	17 (54.8)	58 (43.9)	1,513 (55.5)	2,973 (90.0)	189 (82.9)	25 (83.3)	15 (88.2)	72 (86.7)	3,274 (89.4)
	Current drinker	462 (23.0)	165 (34.1)	23 (35.4)	8 (25.8)	47 (35.6)	705 (25.9)	183 (5.5)	23 (10.1)	4 (13.3)	0 (0.0)	4 (4.8)	214 (5.8)
	Former drinker	374 (18.6)	87 (18.0)	13 (20.0)	6 (19.4)	27 (20.5)	507 (18.6)	148 (4.5)	16 (7.0)	1 (3.3)	2 (11.8)	7 (8.4)	174 (4.8)
Marital status	Married	1,209 (60.1)	343 (70.9)	50 (76.9)	22 (71.0)	94 (71.2)	1,718 (63.0)	1,041 (31.5)	96 (42.1)	13 (43.3)	9 (52.9)	32 (38.6)	1,191 (32.5)
	Unmarried	804 (39.9)	141 (29.1)	15 (23.1)	9 (29.0)	38 (28.8)	1,007 (37.0)	2,263 (68.5)	132 (57.9)	17 (56.7)	8 (47.1)	51 (61.4)	2,471 (67.5)
Brush frequency	Less than once	655 (32.5)	82 (16.9)	13 (20.0)	0 (0.0)	27 (20.5)	777 (28.5)	1,061 (32.1)	32 (14.0)	5 (16.7)	2 (11.8)	14 (16.9)	1,114 (30.4)
	Once or more	1,358 (67.5)	402 (83.1)	52 (80.0)	31 (100.0)	105 (79.5)	1,948 (71.5)	2,243 (67.9)	196 (86.0)	25 (83.3)	15 (88.2)	69 (83.1)	2,548 (69.6)
Hypertension	Yes	763 (37.9)	242 (50.0)	31 (47.7)	16 (51.6)	61 (46.2)	1,113 (40.8)	1,445 (43.7)	109 (47.8)	18 (60.0)	6 (35.3)	48 (57.8)	1,626 (44.4)
	No	1,250 (62.1)	242 (50.0)	34 (52.3)	15 (48.4)	71 (53.8)	1,612 (59.2)	1,859 (56.3)	119 (52.2)	12 (40.0)	11 (64.7)	35 (42.2)	2,036 (55.6)
Diabetes	Yes	170 (8.4)	67 (13.8)	11 (16.9)	3 (9.7)	18 (13.6)	269 (9.9)	319 (9.7)	33 (14.5)	5 (16.7)	1 (5.9)	15 (18.1)	373 (10.2)
	No	1,843 (91.6)	417 (86.2)	54 (83.1)	28 (90.3)	114 (86.4)	2,456 (90.1)	2,985 (90.3)	195 (85.5)	25 (83.3)	16 (94.1)	68 (81.9)	3,289 (89.8)
BMI	<18.5	263 (13.1)	56 (11.6)	5 (7.7)	4 (12.9)	9 (6.8)	337 (12.4)	615 (18.6)	33 (14.5)	2 (6.7)	4 (23.5)	8 (9.6)	662 (18.1)
	18.5–24.9	1,285 (63.8)	287 (59.3)	38 (58.5)	21 (67.7)	68 (51.5)	1,699 (62.3)	1,916 (58.0)	138 (60.5)	17 (56.7)	7 (41.2)	43 (51.8)	2,121 (57.9)
	≥25	465 (23.1)	141 (29.1)	22 (33.8)	6 (19.4)	55 (41.7)	689 (25.3)	773 (23.4)	57 (25.0)	11 (36.7)	6 (35.3)	32 (38.6)	879 (24.0)
Cardiovascular	Yes	483 (24.0)	126 (26.0)	15 (23.1)	5 (16.1)	41 (31.1)	670 (24.6)	796 (24.1)	65 (28.5)	8 (26.7)	2 (11.8)	27 (32.5)	898 (24.5)
	No	1,530 (76.0)	358 (74.0)	50 (76.9)	26 (83.9)	91 (68.9)	2,055 (75.4)	2,508 (75.9)	163 (71.5)	22 (73.3)	15 (88.2)	56 (67.5)	2,764 (75.5)
Sugar frequency	Almost every day	219 (10.9)	64 (13.2)	11 (16.9)	5 (16.1)	24 (18.2)	323 (11.9)	387 (11.7)	22 (9.6)	3 (10.0)	5 (29.4)	15 (18.1)	432 (11.8)
	At least per week or month	635 (31.5)	149 (30.8)	17 (26.2)	10 (32.3)	44 (33.3)	855 (31.4)	952 (28.8)	72 (31.6)	13 (43.3)	4 (23.5)	28 (33.7)	1,069 (29.2)
	Rarely or occasionally	1,159 (57.6)	271 (56.0)	37 (56.9)	16 (51.6)	64 (48.5)	1,547 (56.8)	1,965 (59.5)	134 (58.8)	14 (46.7)	8 (47.1)	40 (48.2)	2,161 (59.0)
ADL capacity	Non-disabled	1,658 (82.4)	445 (91.9)	60 (92.3)	28 (90.3)	114 (86.4)	2,305 (84.6)	2,533 (76.7)	196 (86.0)	24 (80.0)	15 (88.2)	62 (74.7)	2,830 (77.3)
	Disabled	355 (17.6)	39 (8.1)	5 (7.7)	3 (9.7)	18 (13.6)	420 (15.4)	771 (23.3)	32 (14.0)	6 (20.0)	2 (11.8)	21 (25.3)	832 (22.7)

Age and teeth are expressed as mean ± SD. Categorical variables are indicated by the number of people (%)

As shown in Table 2, age, living in eastern China, income, smoking status, drinking status, tooth brushing frequency, ADL status, and daily consumption of green tea (ORs: 1.377, 95%CI: 1.082–1.752) were associated with men having more than 20 teeth. Age, living in central China, education status, health status, brushing frequency, BMI, sugar intake, ADL status, and daily consumption of black tea (ORs: 2.349, 95%CI: 1.028–5.366) were associated with women having more than 20 teeth.

**Table 2:** Adjusted odds ratio and 95% confidence interval of the variables associated with having ≥20 teeth

	Male (*n*=2,725)		Female (*n*=3,662)	
Teeth categorization, n (%)				
≥20 teeth	880 (32.3)		882 (24.1)	
<20 teeth	1,845 (67.7)		2,780 (75.9)	
Variable	Adjusted odds ratios	*P* value	Adjusted odds ratios	*P* value
Age group				
65-69				
70-74	0.579 (0.437-0.765)	**<0.001**	0.557 (0.425-0.731)	**<0.001**
75-79	0.389 (0.291-0.521)	**<0.001**	0.322 (0.242-0.427)	**<0.001**
80-84	0.284 (0.207-0.388)	**<0.001**	0.244 (0.176-0.336)	**<0.001**
≥85	0.118 (0.086-0.162)	**<0.001**	0.114 (0.083-0.157)	**<0.001**
Ethnicity				
Han				
Minority	1.336 (0.956-1.866)	0.090	0.842 (0.600-1.182)	0.320
Residence				
City				
Rural	0.835 (0.684-1.019)	0.076	0.867 (0.717-1.050)	0.144
Geographical region				
Northern China				
Eastern China	0.712 (0.541-0.937)	**0.015**	0.885 (0.675-1.161)	0.378
Central China	1.191 (0.900-1.575)	0.221	1.431 (1.090-1.870)	**0.009**
South-western China	0.862 (0.656-1.131)	0.284	0.889 (0.678-1.166)	0.396
Education level, years				
0				
1-6	1.032 (0.791-1.346)	0.816	1.205 (0.970-1.498)	0.093
>6	1.261 (0.945-1.683)	0.114	1.922 (1.461-2.530)	**<0.001**
Health				
Good				
Average	0.987 (0.803-1.213)	0.901	0.810 (0.664-0.987)	**0.037**
Poor	0.947 (0.691-1.298)	0.734	0.836 (0.630-1.110)	0.216
Household income				
1(poor)				
2	1.181 (0.875-1.592)	0.277	0.920 (0.700-1.210)	0.553
3	1.235 (0.921-1.656)	0.158	1.013 (0.774-1.326)	0.927
4	1.488 (1.066-2.079)	**0.020**	1.067 (0.772-1.476)	0.694
5(wealthy)	1.455 (1.057-2.003)	**0.021**	1.292 (0.956-1.747)	0.095
Smoking habit				
Never				
0–14 years	0.734 (0.582-0.926)	**0.009**	1.004 (0.648-1.557)	0.984
≥15 years	0.605 (0.474-0.771)	**<0.001**	0.695 (0.314-1.539)	0.369
Drinking habit				
Never				
Current drinker	1.376 (1.097-1.726)	**0.006**	0.827 (0.547-1.250)	0.368
Former drinker	1.108 (0.856-1.435)	0.436	1.495 (0.967-2.312)	0.070
Marital status				
Married				
Unmarried	0.819 (0.653-1.027)	0.084	0.827 (0.673-1.017)	0.072
Brush frequency				
Less than once				
Once or more	1.770 (1.389-2.257)	**<0.001**	1.758 (1.370-2.256)	**<0.001**
Hypertension				
Yes				
No	0.856 (0.704-1.040)	0.117	0.960 (0.794-1.160)	0.670
Diabetes				
Yes				
No	0.832 (0.614-1.128)	0.236	0.995 (0.757-1.308)	0.970
BMI				
<18.5				
18.5–24.9	1.045 (0.755-1.445)	0.791	1.477 (1.084-2.013)	**0.014**
≥25	1.147 (0.801-1.642)	0.456	2.106 (1.503-2.950)	**<0.001**
Cardiovascular				
Yes				
No	1.247 (0.995-1.563)	0.056	1.102 (0.890-1.364)	0.373
Sugar frequency				
Almost every day				
At least per week or month	0.879 (0.634-1.217)	0.437	1.219 (0.848-1.753)	0.285
Rarely or occasionally	1.099 (0.810-1.491)	0.543	2.046 (1.456-2.875)	**<0.001**
ADL capacity				
Non-disabled				
Disabled	0.499 (0.350-0.710)	**<0.001**	0.593 (0.433-0.811)	**0.001**
Tea				
No tea				
Green tea	1.377 (1.082-1.752)	**0.009**	1.090 (0.779-1.524)	0.615
Black tea	1.286 (0.733-2.255)	0.381	2.349 (1.028-5.366)	**0.043**
Oolong tea	1.124 (0.500-2.527)	0.777	1.281 (0.386-4.248)	0.685
Scented tea	1.045 (0.687-1.687)	0.837	1.068 (0.611-1.866)	0.818

As shown in Table 3, age, living in rural areas, educational status, smoking status, tooth brushing frequency, ADL status, diabetes status, and daily consumption of green tea (ORs: 1.539, 95%CI: 1.209–1.959) were associated with men having more than 10 teeth. Age, living in central China, educational status, tooth brushing frequency, BMI, sugar intake frequency, ADL status, drinking green tea, and scented tea daily (ORs: 1.447, 95%CI: 1.052–1.991; ORs: 1.948, 95%CI: 1.137–3.340) were associated with women having more than 10 teeth.

**Table 3:** Adjusted odds ratio and 95% confidence interval of the variables associated with having ≥10 teeth

	Male (*n*=2,725)		Female (*n*=3,662)	
Teeth categorization, n (%)				
≥10 teeth	1,357 (49.8)		1,434 (39.2)	
<10 teeth	1,368 (50.2)		2,228 (60.8)	
Variable	Adjusted odds ratios	*P* value	Adjusted odds ratios	*P* value
Age group				
65-69				
70-74	0.570 (0.416-0.783)	**0.001**	0.586 (0.437-0.784)	**<0.001**
75-79	0.355 (0.260-0.486)	**<0.001**	0.353 (0.265-0.470)	**<0.001**
80-84	0.277 (0.200-0.382)	**<0.001**	0.297 (0.219-0.404)	**<0.001**
≥85	0.111 (0.081-0.152)	**<0.001**	0.127 (0.095-0.171)	**<0.001**
Ethnicity				
Han				
Minority	1.214 (0.882-1.670)	0.235	0.790 (0.590-1.057)	0.113
Residence				
City				
Rural	0.823 (0.683-0.991)	**0.040**	0.901 (0.764-1.063)	0.217
Geographical region				
Northern China				
Eastern China	0.824 (0.636-1.067)	0.142	1.072 (0.843-1.363)	0.571
Central China	1.061 (0.812-1.386)	0.666	1.590 (1.252-2.020)	**<0.001**
South-western China	0.933 (0.719-1.210)	0.601	1.045 (0.823-1.327)	0.718
Education level, years				
0				
1-6	0.990 (0.787-1.245)	0.931	1.348 (1.111-1.637)	**0.003**
>6	1.311 (1.011-1.701)	**0.041**	2.015 (1.552-2.616)	**<0.001**
Health				
Good				
Average	1.132 (0.932-1.374)	0.212	0.954 (0.801-1.135)	0.593
Poor	1.010 (0.753-1.356)	0.945	1.027 (0.802-1.315)	0.834
Household income				
1(poor)				
2	1.262 (0.961-1.656)	0.094	1.119 (0.886-1.413)	0.345
3	1.070 (0.818-1.400)	0.621	1.107 (0.878-1.396)	0.391
4	1.323 (0.969-1.808)	0.078	1.233 (0.926-1.641)	0.152
5(wealthy)	1.291 (0.961-1.735)	0.090	1.295 (0.993-1.689)	0.056
Smoking habit				
Never				
0–14 years	0.756 (0.608-0.940)	**0.012**	0.842 (0.586-1.209)	0.351
≥15 years	0.583 (0.460-0.737)	**<0.001**	0.691 (0.357-1.337)	0.272
Drinking habit				
Never				
Current drinker	1.133 (0.910-1.410)	0.263	0.899 (0.638-1.267)	0.544
Former drinker	1.075 (0.843-1.369)	0.560	1.395 (0.960-2.027)	0.080
Marital status				
Married				
Unmarried	0.864 (0.707-1.055)	0.152	0.857 (0.707-1.036)	0.110
Brush frequency				
Less than once				
Once or more	1.561 (1.266-1.924)	**<0.001**	1.641 (1.353-1.991)	**<0.001**
Hypertension				
Yes				
No	0.860 (0.715-1.036)	0.112	0.925 (0.783-1.092)	0.356
Diabetes				
Yes				
No	0.680 (0.497-0.932)	**0.017**	1.090 (0.840-1.416)	0.515
BMI				
<18.5				
18.5–24.9	0.974 (0.740-1.283)	0.854	1.523 (1.202-1.930)	**<0.001**
≥25	1.092 (0.794-1.502)	0.589	1.669 (1.269-2.195)	**<0.001**
Cardiovascular				
Yes				
No	1.175 (0.947-1.458)	0.142	1.022 (0.845-1.237)	0.820
Sugar frequency				
Almost every day				
At least per week or month	0.950 (0.706-1.278)	0.736	1.297 (0.971-1.732)	0.079
Rarely or occasionally	1.026 (0.775-1.357)	0.859	1.631 (1.243-2.140)	**<0.001**
ADL capacity				
Non-disabled				
Disabled	0.527 (0.399-0.696)	**<0.001**	0.699 (0.556-0.879)	**0.002**
Tea				
No tea				
Green tea	1.539 (1.209-1.959)	**0.001**	1.447 (1.052-1.991)	**0.023**
Black tea	1.447 (0.797-2.624)	0.225	2.292 (0.970-5.415)	0.059
Oolong tea	0.701 (0.318-1.546)	0.378	0.762 (0.244-2.381)	0.641
Scented tea	0.854 (0.565-1.291)	0.454	1.948 (1.137-3.340)	**0.015**

In the model of the remaining 20 teeth (Table 4), we found that drinking tea significantly increased the number of teeth only in the population that brushed their teeth. In the daily brushing group, green tea had a significant beneficial effect on increasing the number of teeth in men (ORs: 1.401, 95%CI: 1.077–1.821) and black tea had a significant beneficial effect in women (ORs: 2.653, 95%CI: 1.088–6.468).

**Table 4:** Adjusted odds ratio and 95% confidence interval of the tea types associated with having ≥20 teeth

Variable		Male (*n*=2,725)	Female (*n*=3,662)
		Number of teeth (≥20)	Number of teeth (≥20)
Brush less than once	No tea		
	Green tea	1.115 (0.569–2.185)	0.864 (0.206–3.626)
	Black tea	0.510 (0.128–2.119)	0.515 (0.030–8.933)
	Oolong tea	NA	NA
	Scented tea	0.478 (0.151–1.515)	0.989 (0.178–5.499)
Brush once or more	No tea		
	Green tea	1.401* (1.077–1.821)	1.079 (0.762–1.529)
	Black tea	1.578 (0.833–2.990)	2.653* (1.088–6.468)
	Oolong tea	1.155 (0.513–2.601)	1.734 (0.490–6.137)
	Scented tea	1.212 (0.766–1.919)	1.034 (0.568–1.884)

Adjusted for age, ethnicity, residence, geographical region, educational level, health, household income, smoking habit, drinking habit, marital status, hypertension, diabetes, BMI, cardiovascular, sugar frequency and ADL capacity

^*^p<0.05

In the model of the remaining 10 teeth shown in Table 5, we found that tea consumption had a significant effect on increasing the number of teeth only in the population that brushed their teeth. In the daily brushing group, consumption of green tea and black tea had significant beneficial effects on increasing the number of teeth in men (ORs: 1.748, 95%CI: 1.329–2.299; ORs: 2.133, 95%CI: 1.031–4.416), whereas that of green tea, black tea, and scented tea had significant beneficial effects in women (ORs: 1.474, 95%CI: 1.044–2.081; ORs: 3.212, 95%CI: 1.181–8.736; ORs: 2.320, 95%CI: 1.270–4.239).

**Table 5:** Adjusted odds ratio and 95% confidence intervals of the tea type associated with having ≥10 teeth

Variable		Male (*n*=2,725)	Female (*n*=3,662)
		Number of teeth (≥10)	Number of teeth (≥10)
Brush less than once	No tea		
	Green tea	0.705 (0.393–1.264)	0.982 (0.368–2.619)
	Black tea	0.373 (0.100–1.390)	0.125 (0.008–1.877)
	Oolong tea	NA	NA
	Scented tea	0.389 (0.141–1.072)	0.859 (0.199–3.713)
Brush once or more	No tea		
	Green tea	1.748** (1.329–2.299)	1.474* (1.044–2.081)
	Black tea	2.133* (1.031–4.416)	3.212* (1.181–8.736)
	Oolong tea	0.755 (0.341–1.669)	0.996 (0.289–3.429)
	Scented tea	0.978 (0.614–1.557)	2.320** (1.270–4.239)

Adjusted for age, ethnicity, residence, geographical region, educational level, health, household income, smoking habit, drinking habit, marital status, hypertension, diabetes, BMI, cardiovascular, sugar frequency and ADL capacity

^*^p<0.05

^**^p<0.01

## Discussion

In this study, we found that daily tea consumption over a long period of time may be significantly related to the number of teeth remaining in older adults. Previous studies have reported that diet and drinking water were significantly associated with a reduction in the number of teeth among older adults in China [24]. We stratified the total population according to sex and toothbrushing frequency and performed multivariate logistic regression analysis using two models: threshold of 10 remaining teeth and 20 remaining teeth. Among all tea types, green tea contains the highest amount of catechins [27, 28]. Sex was significantly associated with tooth loss, which is also consistent with previous findings and may be attributed to sex-related differences in genetics and hormone production as well as sex-based cultural influences [29–32]. There are sex-related differences in the incidence of many chronic diseases, which affect general health and further impact oral health [21, 33]. Green tea can improve oral health in both men and women, whereas black tea can improve oral health in women and those with the habit of brushing teeth. Moreover, scented tea only improves oral health in women with more than 10 teeth. However, our findings did not indicate that oolong tea improved oral health, which may be attributed to the small sample size. Our findings are consistent with those of previous reports, regardless of the type of tea beneficial to oral health [16, 34, 35].

Age is a major risk factor for tooth loss [36, 37], which is consistent with our findings. Additionally, we found that geographical factors may be associated with oral health, which is consistent with previous findings [38]. Moreover, men living in rural areas have a higher prevalence of severe oral tooth loss (<10 teeth), which is consistent with our findings using the same dataset analysis [23]. This may be attributed to the poor geographical accessibility of dental services for men in rural areas, and delayed treatment may contribute to this trend [39–41]. Furthermore, this may be due to geographical differences in drinking water sources and fluoride levels; however, there is no clear evidence indicating that fluoride levels vary widely across regions in China [24]. We also found that marital status was associated with a reduction in tooth count even with daily brushing, which could be attributed to social networking, similar to that reported in previous Japanese studies [42]. Compared with men, women experienced an obvious effect of education status on improvement in oral health. This may be due to sex-related differences in educational status among the Chinese older adults, with the illiteracy rate being 62.7% in women and 23.0% in men. Household income had a significant relationship with maintaining good oral health but not with severe tooth loss, which may be attributed to the fact that there are too many factors for severe tooth loss and the reduced influence of income factors on oral health [43].

We observed a significant adverse effect of smoking on oral health among men, which is consistent with previous findings [44–46]. Among men with a current drinking habit, there was an improvement in oral health, which is inconsistent with previous findings [44, 47]; however, two Japanese studies showed that, among men, current drinkers had a significantly lower risk of having <20 teeth [48, 49]. It is well-known that the frequency of teeth brushing is strongly correlated with oral health [50], which is consistent with our findings. Increasing the frequency of tooth brushing can significantly improve oral health. Another study based on this data found that the prevalence of chronic diseases significantly decreased with age [51]. In our study, hypertension and diabetes revealed an association between age and the risk of tooth loss. After adjusting for age and other risk factors in the logistic function, this correlation was no longer statistically significant [21].

Sugar is crucially involved in the occurrence of dental caries [52] and seldom or occasional sugar intake significantly improved oral health in this study, which is also consistent with the theory that less sugar consumption benefits oral health. The oral health of people with disabilities having ADL abilities significantly declined, which may be attributed to the inconvenience of visiting a doctor. To address this issue, we should provide them with more assistance, such as dental home visits [53].

Our study has several advantages, including a large sample size and CLHLS being a well-designed project with relatively reliable data. To the best of our knowledge, this is the first study to investigate the relationship between tea types and oral health. However, this study has some limitations. First, because this was a cross-sectional study, there are many potential residual confounding factors, which may have resulted in bias. Although we selected people who had been drinking the same tea for 5 consecutive years through questionnaire surveys, we could not infer a causal relationship between tooth loss and tea drinking habits [54]. Second, the questionnaire only collected data on the frequency of tea consumption rather than the amount of tea consumed each time. Third, the number of natural teeth in the CLHLS was self-reported, which may introduce measurement error. However, Western and Asian populations in previous studies indicated that self-reports were considered valid alternatives to clinical measures to estimate tooth counts in adult population [55, 56]. Finally, the study population comprised a very old sample, with participants generally being older than the older adult population in China. Therefore, the findings may not be applicable to other populations.

Future studies should consider combining epidemiological data with basic research (i.e., antibacterial experiments) for each type of tea to verify the effects of tea consumption on oral health. In future public health recommendations and in clinical practice, health professionals should pay careful attention to dental status and the type of tea consumed by sex-specific groups. Additionally, the importance of brushing one’s teeth and active promotion of oral health awareness should be emphasized to improve oral health outcomes.

## Conclusions

Among the elderly population in China, long-term consumption of green tea by men and black tea by women may be significantly associated with maintaining functional dentition (≥20 teeth). For men, long-term consumption of green tea, and for women, long-term consumption of both green tea and scented tea, may be linked to avoiding severe tooth loss (≥10 teeth). In the daily toothbrushing group, in addition to confirming the above conclusions, long-term consumption of black tea may be associated with avoiding severe tooth loss for both men and women. However, tea consumption alone did not have an impact on oral health without good brushing habits.

## Data Availability

This study was based on datasets from the Chinese Longitudinal Healthy Longevity Survey (CLHLS) conducted in longevity areas. The CLHLS data can be publicly obtained through Peking University Open Research Data (https:// opendata.pku.edu.cn/dataverse/CHADS).

## Abbreviations

CI: Confidence interval
CLHLS: Chinese Longitudinal Healthy Longevity Survey
ORs: Odds ratio
SD: Standard deviation
ADL: Activities of daily living
BMI: Body mass index
